# Metallo-β-Lactamases and Aptamer-Based Inhibition

**DOI:** 10.3390/ph4020419

**Published:** 2011-02-18

**Authors:** Sara R. Schlesinger, Mieke J. Lahousse, Taylor O. Foster, Sung-Kun Kim

**Affiliations:** Department of Chemistry and Biochemistry and the Institute of Biomedical Studies, Baylor University, Waco, TX 76798, USA

**Keywords:** metallo-β-lactamase, aptamer, SELEX, inhibitor, *Bacillus cereus*

## Abstract

An evolution of antibiotic-resistant bacteria has resulted in the need for new antibiotics. β-Lactam based drugs are the most predominantly prescribed antibiotics to combat bacterial infections; however, production of β-lactamases, which catalyze the hydrolysis of the β-lactam bond of this class of antibiotics, by pathogenic bacteria such as *Bacillus cereus*, are rendering them useless. Some inhibitors of β-lactamases have been found, but there are no inhibitors against a class of β-lactamases known as metallo-β-lactamases, and it has been reported that the number of bacteria that produce metallo-β-lactamases is on the rise. Finding inhibitors of metallo-β-lactamases is thus an urgent necessity. One way to approach the problem is by employing the combinatorial method SELEX. The SELEX method is significant in discovering and producing new classes of inhibitors, as well as providing insight into the development of these inhibitors and paves the way for future aptamer applications that further novel drug discovery.

## 1. Introduction

**Figure 1:**
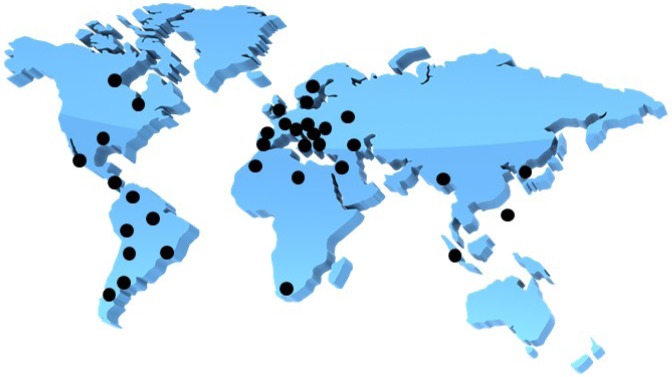
The global distribution of NDM-1 (adopted from reference [2]).

A male patient who lived in Sweden often traveled to India and was found to be infected with an extended-spectrum β-lactamase (ESBL)-producing *Klebsiella pneumoniae* [1]. The β-lactamase isolate from New Dehli has been named NDM-1 or bla_NDM-1_, which stands for New Dehli Metallo-β-lactamase. The emergence of the NDM-1 plasmid is a disturbing development because it carries resistance to not only β-lactams, but also macrolides, aminoglycosides, rifampicin, sulfamethoxazole and aztreonam [1]. This multi-drug resistant *Klebsiella pneumoniae* containing NDM-1 has thusly been named a ‘superbug’. NDM-1 is mainly found in *K. pneumoniae*, but recently other bacteria such as Enterobacteriaceae and *Acinetobacter baumannii* display NDM-1 activity as a result of the genetic plasticity of the plasmid that carries the genes responsible for producing the resistant mechanisms [1]. NDM-1 has spread throughout the world, including the USA, UK, Canada, Australia, continental Europe, Eastern Europe, the Middle East, Africa and South East Asia (Figure 1) [1,2]. To overcome the issue of bacterial antibiotic resistance, one solution is to discover inhibitors of metallo-β-lactamases (MBLs). Industries and academia have therefore focused intensively on discovering inhibitors of MBLs; however, no compounds are hitherto approaching phase 1 clinical trials. Hence, it is an exceedingly pressing issue when we consider the more than eight years typically necessary to approve new inhibitors as being safe enough to appear in pharmaceutical markets. The recent discovery of aptamer-based inhibitors of MBL from *Bacillus cereus* is interesting enough to catch our attention. In this review, we will discuss β-lactamases, development of aptamers against MBL from *B. cereus*, and aptamer-based inhibition tests of MBL.

## 2. β-Lactamases

β-Lactamases (β-lactam hydrolyases, EC 3.5.2.6) are highly efficient enzymes that inactivate β-lactam antibiotics by catalyzing the hydrolysis of the four-membered β-lactam ring of the antibiotics. So far, more than 700 β-lactamases have been identified, and are classified into four groups, A–D [3]. The class A, C, and D β-lactamases use a serine residue in the active site as a nucleophile to attack substrates, and these enzymes are called serine-β-lactamases [4,5]. The class A β-lactamases tend to attack penicillins, while the class C β-lactamases have a tendency to attack cephalosporins [4,5]. In the case of class D β-lactamases, the serine in the active site prefers to hydrolyze oxacillin [4,5]. However, the β-lactamases in the class B require one or two zinc ions for their full catalytic activity, and these enzymes are therefore called metallo-β-lactamases (MBLs) [4,5]. The enzymes have a wide range of substrate specificity [6].

The first MBL was found in an innocuous strain of *Bacillus cereus*, and MBL-mediated resistance has rapidly spread to pathogenic bacteria [4]. These MBLs can be categorized into three subgroups, B1–B3. The B1 enzymes require one or two zinc ion(s) for the activity, where the tightly bound zinc is referred to as Zn_1_ and the less tightly bound zinc is called as Zn_2_ [4,5]. In the case of Zn_1_, a tetrahedral geometry is coordinated by three histidines and one solvent molecule [4]. A distorted trigonal bipyramidal geometry is the coordination of Zn_2_ by three amino acids (His, Cys, Asp), a water molecule and a solvent (e.g., H_2_O and glycerol) that also serves as a ligand to Zn_1_ [4]. For the B2 enzymes, a salient feature is that the B2 enzymes require only one zinc ion for their full activity [4]. Lastly, the B3 enzymes require two zinc ions for the full activity, but the difference between B1 and B3 enzymes is that the Zn_2_ ion in B3 is coordinated to another His in place of Cys [4]. The most notable plasmid- and chromosome-mediated MBLs today are listed in Table 1, along with their classification and the bacteria they have been identified in. It should be mentioned here that typically plasmid-encoded β-lactamases that are more transferable may confer resistance to expanded-spectrum β-lactams, whereas chromosome-encoded β-lactamases may confer narrow-spectrum β-lactams. This infers that the plasmid-encoded β-lactamases draw more attention than chromosome-encoded β-lactamases.

To protect β-lactam antibiotics, inhibitors of β-lactamases are needed. Mechanism-based irreversible inhibitors, such as clavulanic acid, sulbactam, and tazobactam, have been used to inactivate serine-β-lactamases, which are metal-independent-β-lactamases [5]. The co-administration of an antibiotic with one of these inhibitors has been commercially applied to cure β-lactam antibiotic resistant bacterial infections. It has been demonstrated that some potential inhibitors for serine-β-lactamase have the ability to inactivate MBLs [7]. However, none of the commercially available inhibitors of metal independent-β-lactamases are effective against MBLs.

**Figure 2:**
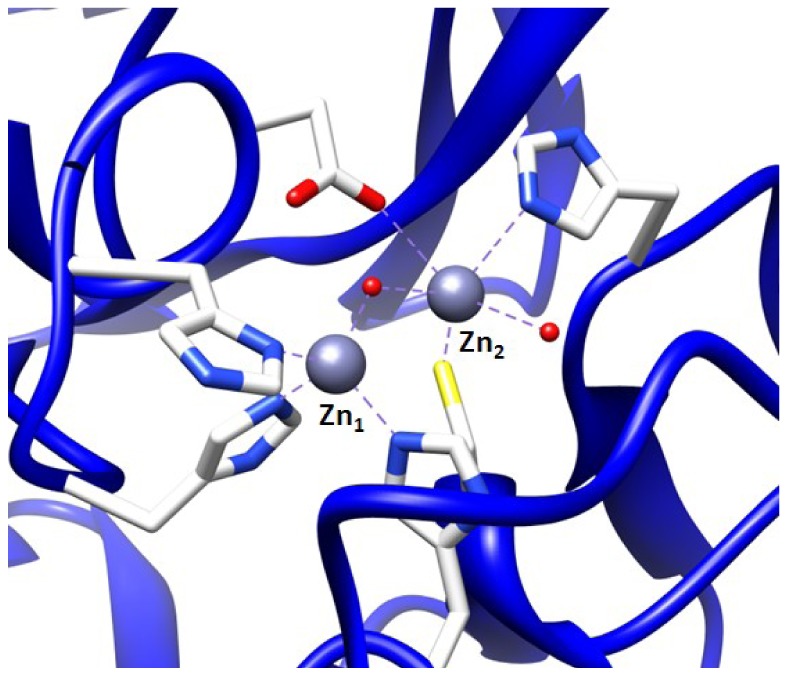
X-ray structure of BcII (PDB: 1BC2). The sulfur of the cysteine residue in active site of BcII is colored in yellow.

To find inhibitors of the metallo-β-lactamase BcII from *B. cereus*, whose active site is shown in Figure 2, the combinatorial approach SELEX was employed. Through combinatorial chemistry a wide variety of compounds can be effectively tested for promising drug activity. Combinatorial methods have been widely adopted by biotechnology and pharmaceutical companies over the past ten years.

## 3. SELEX Technology

**Figure 3:**
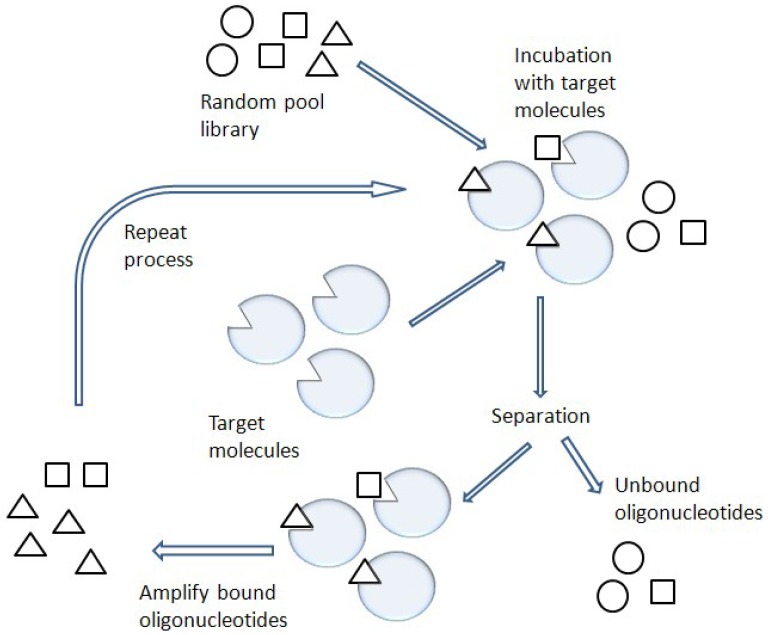
Scheme of SELEX. Target molecules are incubated with a library of oligonucleotides. The separation occurs by the degree of binding affinities of the oligonucleotides. The bound oligonucleotides are amplified by PCR. The iterative process is performed to find functional aptamers.

One of the most promising combinatorial chemistry techniques is known as SELEX [8] (Figure 3). This SELEX method is also known as *in vitro* selection or *in vitro* evolution, allowing the simultaneous screening of a large number of nucleic acid molecules.

Functional nucleic acid molecules are selected from the mainly non-functional pool of oligonucleotides by column chromatography or other selection techniques such as a gel shift assay [9-11]. The functional nucleic acids are called aptamers, which are usually short single-stranded (ss) nucleic acids such as ssDNA and RNA [9]. Many of the selected aptamers display affinities for their target comparable to those observed for monoclonal antibodies. However, unlike antibodies, facile modification of the selected aptamers can improve their binding to target molecules and enhance the stability of the aptamers against nuclease activity under physiological conditions [12].

The application of aptamers has been significant in the medical and pharmaceutical research fields. A recent example of a commercial product developed using SELEX technology is an aptamer against vascular endothelial growth factor (VEGF). In fact, this is the only commercially available aptamer-based therapy. The anti-VEGF aptamer blocks vessel growth and inhibits neovascularization [13,14] with very high affinity (dissociation constant, *K*_d_ = 50 pM) [15] (this aptamer affinity is as good as the antibody affinity (*K*_d_ = 54 pM) for VEGF [16]). The aptamer tightly binds to abnormally over-expressed VEGF and thus the binding reaction from VEGF to a receptor cannot proceed. This aptamer, known as Macugen^®^, was approved by the Food and Drug Administration (FDA) in 2004 and is currently used to cure age-related macular degeneration [17,18].

Although the anti-VEGF aptamer is based on RNA SELEX, recently we reported that ssDNA SELEX for the *B. cereus* metallo-β-lactamase was used to find ssDNA aptamers which act as inhibitors of the enzyme, thus providing the possibility of an antibacterial drug against this specific β-lactam resistant bacterial infection [19]. Other potential drug candidates using ssDNA SELEX technology have been developed. For example, thrombin, a protein that serves as essential role in regulation of the coagulation pathway in human, has been targeted for the development of ssDNA aptamers; an ssDNA aptamer of thrombin has been identified and shows a very promising anticoagulant drug activity [20,21]. Anti-inflammatory aptamers for L-selectin [20], viral infection prevention aptamers for Hemagglutinin from the influenza virus [22], and anti-progressive renal disease aptamers for platelet-derived growth factor [23] have been developed using ssDNA SELEX technology as well. Except for the aptamers of *B. cereus* metallo-β-lactamase, other aptamers functions by tightly binding to target molecules and interfering with the target molecules' next binding step. In the following section, introducing true enzymatic inhibition to *B. cereus* metallo-β-lactamase in an aptamer-based inhibition manner will draw our attention as to how we can screen for enzyme inhibiting aptamers.

## 4. SELEX for *B. Cereus* Metallo-β-Lactamase Using ssDNA

Previously, the metallo-β-lactamase BcII from *B. cereus*, a class B MBL, as a target molecule to find aptamers using SELEX technology has been studied. *B. cereus* was chosen because it is a pathogen that causes food poisoning and because the three-dimensional X-ray structure of BcII has been resolved (Protein Data Bank entry code: 1BC2), which increases the chances of improving our understanding of binding between potential inhibitors and BcII. This project was very successful, and this work was recently published [19]. This study had three aims, which were: (a) to find aptamers that inhibit BcII; (b) to determine inhibition values of the found aptamers by kinetic analyses, and explore the binding relationship between the aptamers and metal ions in the active site of the enzyme; (c) and to test the specificity of the aptamers and the growth inhibition of *B. cereus* by the aptamers in combination with an existing antibiotic.

### 4.1. Identification of Aptamers that Can Inhibit the Target Enzyme

**Figure 4:**
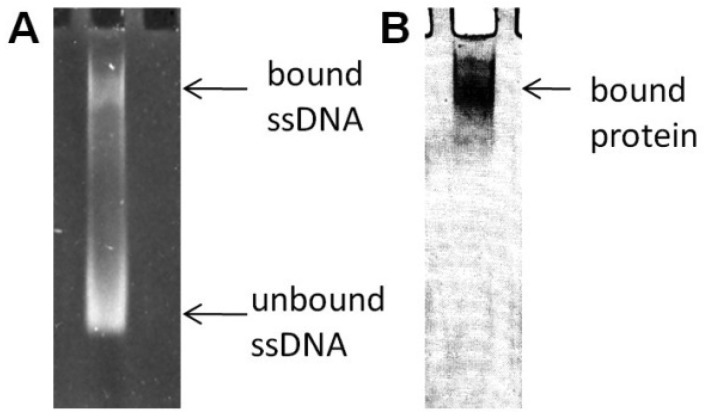
Electrophoretic Mobility Shift Assay. (**A**). The typical separation between bound oligonucleotides and unbound oligonucleotides. The 6% polyacrylamide gel was stained by ethidium bromide; (**B**). The gel was stained by Coomassie Brilliant Blue R-250 to confirm the complex form of the target protein and ssDNA (taken from reference [24]).

In the previously reported literature [19], the SELEX method was used to screen aptamers against the *B. cereus* MBL. Randomly degenerated ssDNA and the target enzyme were incubated for an appropriate period of time. A complex formation between ssDNA and the MBL was confirmed on a native gel, as shown in Figure 4. In an effort to confirm the complex, the sample was loaded in two different gel lanes on the same native gel. The gel was soaked in an ethidium bromide solution to illuminate the bound ssDNA to the enzyme after completion of the electrophoresis; the same gel was stained in Coomassie Blue to identify the existence of the enzyme. In the native gel, any non-complexed enzyme would not enter the gel due to its high isoelectric point value, but the complex migrates toward the gel bottom because it contains anionic ssDNA. During the iterative process of SELEX, to sequester tightly bound aptamers from both unbound and non-specific bound ssDNAs, more stringent selection was exerted by an increasing NaCl concentration. A typical gel after loading the sample under such conditions clearly showed two bands by ethidium bromide staining; that is, the enzyme:ssDNA complex and the unbound ssDNA (Figure 4).

The purpose was to identify aptamers that inhibit BcII, thus it was of great importance that enzyme inhibition assays be carried out during the SELEX rounds [19]. To test whether ssDNA could bind and inhibit BcII, the selected ssDNA pools were subjected to inhibition assays and only one oligonucleotide was identified after DNA sequencing, which was 30 residues in length. To understand the binding between the aptamer and the target molecule, it is necessary to recognize the importance of secondary structures formed by the ssDNA. The secondary structure of the found sequence was predicted by MFold, a secondary structural nucleic acid prediction program [25]. This program uses energy rules to predict optimal secondary structures for an RNA molecule as well as an ssDNA molecule [26,27]. By using the MFold program, two different secondary structures of the aptamer (a 30 residue ssDNA) were predicted. The two different structures commonly contained a 10 residue stem-loop structure, therefore this sequence was considered to be important to enzyme inhibition. Hence, further investigation was carried out using the 10 residue ssDNA.

### 4.2. The Determination of IC_50_ and Kinetic Parameters

The IC_50_value, the concentration of inhibitor necessary to inhibit 50% of the enzyme's activity, for the aptamers were determined by measuring the rate of enzymatic hydrolysis of a substrate [19]. The IC_50_ of the 30-oligonucleotide ssDNA was 1.2 nM, and the IC_50_ of the 10 residue ssDNA was also 1.2 nM [19]. These results indicate that the 10 residue ssDNA was the crucial sequence for enzyme inhibition. The remainder of the sequence was tested to determine whether other portions of the 30 residue ssDNA contributed to the inhibition, but no inhibition was found [19]. This data provides evidence to support the idea that the 10 residue ssDNA is responsible for the observed inhibition.

The steady state enzyme kinetic data of the inhibition of BcII were obtained by various concentrations of the 10 and 30 residue ssDNAs [19]. The inhibition data showed reversible, mixed inhibitions for both the 10 and 30 residue ssDNAs, with dissociation constants for the enzyme-inhibitor complex (*K_i_*) and the enzyme-substrate-inhibitor (*K_i_′*) in the nanomolar range, demonstrating that inhibition by those ssDNA aptamers is very effective. Previously, possible inhibitors had been developed by rational-drug approach with *K_i_* values in the micromolar range [7]. This comparison strongly suggests that the findings (10 and 30 residue ssDNAs) are of great value in further investigating the binding of the aptamers to the target molecule.

### 4.3. The Test of the Effect of the Aptamer Binding to the Enzyme by Co^2+^ Reconstitution

It is possible to prepare an apoenzyme of a metallo-β-lactamase and reconstitute the original enzymatic activity by the addition of cobalt (II) sulfate [28]. Given that the Co^2+^-reconstituted MBL can provide a signature feature in the UV-Vis range, the reconstituted enzyme is then useful in investigating the binding relationship between the metal ion and the ssDNA aptamer. The preparation of Co^2+^-reconstitution was successful in BcII from *B. cereus* and thus was used for UV-Vis spectroscopy [19]. The observation of the visible electronic spectra of the Co^2+^ reconstituted enzyme in the absence and the presence of the 10 residue ssDNA showed significant differences [19].The changes in the spectrum observed by addition of the ssDNA at the cysteine-Co^2+^ charge transfer band were observed particularly at a wavelength of 347 nm. Similar changes were observed in the presence of an excess of substrates such as cephalosporin C [28,29]. Therefore, the change at 347 nm indicates that the cysteine thiol group located in the active site of the enzyme, where the cysteine thiol group serves as a ligand to the zinc ion at the Zn_2_ position (Figure 2), was affected by the ssDNA addition. Thus, the observation of the change of active site by the addition of the ssDNA aptamer supports the proposal that the ssDNA aptamer's binding to the enzyme elicits inhibition of the metallo-β-lactamase.

### 4.4. Specificity

One of the most important aspects of inhibitor design is that the inhibitors should be specific to the enzyme. To test the specificity, porcine carboxypeptidase A, a zinc metalloenzyme, and *B. cereus* serine-β-lactamase were used [19]. The addition of ssDNA aptamers (both 10 and 30 residue ssDNAs) to porcine carboxypeptidase A exerted no influence on its enzymatic activity, thus suggesting that the ssDNA aptamer do not affect all zinc binding sites indiscriminately [19]. The fact that addition of the ssDNA aptamer to the *B. cereus* serine-β-lactamase had no impact on the enzyme activity [19] provides additional evidence for the specificity of the ssDNA aptamer to the metallo-β-lactamase. In addition, the observation that the *K*_i_ value of the Co^2+^-reconstituted enzyme by the 10 residue ssDNA was almost 4 orders of magnitude higher than that of the normal metallo (Zn^2+^)-β-lactamase further demonstrates the exquisite specificity of the ssDNA aptamer in that the DNA is able to distinguish between the zinc and cobalt forms of the enzyme, even though both enzyme forms possess the same enzymatic activity.

### 4.5. Inhibition Tests in Bacteria

To explore the possibility that co-administration of the found aptamer and a β-lactam antibiotic have the capacity to negatively impact the growth of β-lactam antibiotic resistant bacteria, the combination of the 10 residue ssDNA with a β-lactam antibiotic, cephalexin, was applied to *B. cereus* 5/B/6, which produces the MBL [19]. The bacterial growth was suppressed over a 20 h period at 30 °C. As control experiments, either the antibiotic alone or the aptamer alone was used, and those had no effect on the bacterial growth; however, to obtain cell death, in the presence of 5 μM cephalexin, 75 μM of the 10 residue aptamer was needed [19]. This indicates that some nuclease activities may degrade the nucleotide aptamer under cell culture conditions. This observation suggests that modification of the aptamer is necessary to retain its strong potency as an enzyme inhibitor.

## 5. Future Studies

In this review, we describe the potential of an aptamers-based method using SELEX technology to discover novel inhibitors of the metallo-β-lactamase BcII from *Bacillus cereus*. Although we should mention that there is a possibility that the aptamers-based inhibitors to BcII would be limited to chromosome-encoded β-lactamases that may structurally differ from plasmid-encoded β-lactamases, there has been success in finding potential inhibitors to BcII in an aptamers-based inhibition fashion. To further improve the inhibition for those ssDNA aptamers, modifications of the aptamers are likely needed so that they can survive degradation by nucleases and have more potential as novel drug candidates. For example, phosphorothioate modification, which takes place at a non-bridging oxygen in the phosphate backbone at the 5′-and/or 3′-end of the aptamers, would be a good choice. This modification leads to resistance to degradation from endo- and exonuclease cleavage. Another issue in developing oligonucleotide therapeutics is the relatively short half-lives of the aptamers in the body. To overcome this problem, conjugation to polyethylene glycol (PEG) increases the molecular weight of the aptamer and extends its elimination half-life by slowing renal filtration and distribution from the central compartment. This PEG conjugation has emerged as an effective strategy to extend the circulating half-life [30]. For instance, the use of 40 kDa PEG conjugated to an aptamer was reported to extend the serum half-life time to a significant extent [31]. These strategies to optimize aptamer therapeutics are important in combination with SELEX technology to identify a new class of drug candidates that may be used in the battle against the proliferation of antibiotic-resistant bacteria.

## Figures and Tables

**Table 1:** Description of most common plasmid- and chromosome-mediated MBLs, their classification, and the bacteria in which they are found.

**Name**	**Class**	**Bacteria**	**Encoding**
IMP	B1	*Pseudomonas aeruginosa*, *Acinetobacter baumannii*, *Serratia marcesens*, *Enterobacter aerogenes*	Plasmid
VIM	B1	*Pseudomonas aeruginosa*, *Acinetobacter baumannii*, *Klebsiella pneumoniae*, *Enterobacter aerogenes*	Plasmid
SPM	B1	*Pseudomonas aeruginosa*	Plasmid
GIM	B1	*Pseudomonas aeruginosa*	Plasmid
AIM	B3	*Pseudomonas aeruginosa*	Plasmid
SIM	B1	*Pseudomonas aeruginosa*, *Acinetobacter baumannii*	Plasmid
NDM	unknown	*Klebsiella pneumoniae*	Plasmid
DIM	B1	*Pseudomonas stutzeri*	Plasmid
KHM	unknown	*Citrobacter freundii*	Plasmid
BcII	B1	*Bacillus cereus*	Chromosome
Bla2	B1	*Bacillus anthracis*	Chromosome
CcrA	B1	*Bacteroides fragilis*	Chromosome
ImiS	B2	*Aeromonas sobria*	Chromosome
L1	B3	*Stenotrophomonas maltophilia*	Chromosome
